# Therapy-facilitated integration responsibility: qualitative interviews with refugee psychotherapy clients in Germany

**DOI:** 10.1186/s12888-025-07716-0

**Published:** 2026-01-12

**Authors:** Flurina Potter, Anke Richter, Katalin Dohrmann, Anselm Crombach

**Affiliations:** 1 Department of Psychology, University of Konstanz, Konstanz, Germany; 2 Vivo International, Konstanz, Germany; 3 Department of Psychology, Saarland University, Saarbrücken, Germany

**Keywords:** Refugees, Mental health, Psychotherapy, Qualitative research, Grounded theory, Integration, Health care, Germany

## Abstract

**Background:**

Many refugees experience substantial pre-, peri- and post-migration stress resulting in high prevalence rates of mental disorders. Additionally, refugees face significant challenges integrating into their host society. Even though access to psychotherapy for refugees might impact the integration process beneficially, research on refugees’ perspectives on psychotherapy, integration and the interaction with emotional distress is limited. This study explores refugees’ perspectives on how psychotherapy may influence their integration process and enhance their psychological well-being.

**Method:**

Six in-depth, problem-centered interviews were conducted with refugees after they had completed psychotherapy in Germany one to two years earlier. The male interviewees, aged between 23 and 44 years, were from Afghanistan (*n* = 1), Gambia (*n* = 3), Senegal (*n* = 1) and Turkey (*n* = 1). The interviews were conducted in English or German and lasted between 35 and 90 min. The data was analyzed with Constructivist Grounded Theory.

**Results:**

The qualitative analysis led to the development of the framework Therapy-Facilitated Integration Responsibility. This framework identified therapy experiences, especially the Therapeutic Relationship, Opening Up and Being Guided, as crucial for taking on integration measures such as language acquisition, employment, and combating loneliness, ultimately fostering well-being in the host country. Therapy helped the interviewees to overcome a Burdened Integration Responsibility, which was strongly influenced by Stressors Before Arrival, Post-Migration Stressors and Everyday Loneliness.

**Conclusion:**

This study gives insight into the interplay of refugees’ emotional distress and integration. It underlines the need to promptly address refugees’ emotional distress and loneliness to support them tackling integration measures soon after their entry into the host country.

**Clinical trial number:**

Not applicable.

**Supplementary Information:**

The online version contains supplementary material available at 10.1186/s12888-025-07716-0.

## Background

Germany has become one of the major host countries for refugees and in 2023 hosted around 2.6 million refugees [1]. Numerous studies have demonstrated that the substantial pre- and peri-migration stress refugees experience causes a high prevalence of mental disorders [2–4]. Moreover, research has shown that post-migration stress can exacerbate trauma-related symptoms stemming from pre- and peri-migration stressors, thereby impeding successful integration [3–6]. Nevertheless, refugees in Germany face many barriers to accessing mental health care services and only a few receive the recommended treatment [7, 8]. Additionally, hostility and hatred towards refugees have increased [9, 10]. As integration remains challenging for states, societies, and health care systems [4, 11], this study focused on refugees’ perspectives on psychotherapy, integration and their influence on emotional distress.

In this study, we define **integration** as having the knowledge and skills to live successful and healthy lives in the host country [12]. Harder [12] proposed six different integration dimensions: psychological well-being, economic stability, political participation, social connections, language proficiency, and the ability to navigate public institutions. Refugees in Germany face several integration barriers: employment bans due to asylum procedures [13], living in refugee shelters has been shown to lead to less social integration [14], and long waiting lists or illiteracy may complicate access to language and integration courses [15]. While ideological ideas in Germany regarding what a “good” or “deserving” refugee should be like have been described as making “efforts in language learning, cultural integration, and economic contributions” [16], research has emphasized the reciprocity and duality of the integration process [14–17]. The welcoming host country may play a crucial role in soliciting refugees’ integration and well-being [18] and a lack of social connections may lead to loneliness, helplessness and anxiety, as well as to a stagnation of language skills [14]. Phillimore [19] connected refugees’ pre-, peri-, and post-migration stress to their inability to socialize, and the interviewed refugees reported frustration and anxiety regarding the social integration expected from them. This result aligns with qualitative studies in which interviewed refugees have described their social connectedness as quite low [20]. Furthermore, these studies revealed and critiqued an integration narrative presenting language learning and employment as individual efforts [15]. Taking on integration measures can be further complicated by cognitive changes caused by post-traumatic stress disorder [21] and other psychological disorders [4]. In line with these conclusions, Walther et al. [22] found, in a representative survey on refugee adults in Germany, that their psychological distress was linked to integration difficulties, particularly with employment and the attendance and successful completion of language and integration courses. In a subsequent qualitative study, Walther et al. [23] interviewed refugees and concluded that psychological distress due to past experiences, but also due to residence and integration law requirements in the host country, resulted in a reduced ability to pursue integration activities.

There are several studies focusing on therapists’ perspectives on psychotherapy with refugee clients. The majority highlight challenges such as dealing with interpreters in therapy [24], bureaucracy [25], and differing explanatory models for psychotherapy [24, 26, 27]. On the other hand, a few qualitative studies with refugees highlighted the importance of the interpersonal connection with a caring therapist [28] and the therapeutic relationship [29]. In a study with unaccompanied minors receiving Narrative Exposure Therapy (NET), Said et al. [30] found that the interviewees perceived NET as helpful for understanding one’s life better and starting more positively into adulthood. Said [30] argued that due to the loss of parents and caregivers, particularly adolescents required a stable therapeutic relationship. The qualitative meta-synthesis by Khairat et al. [31] identified five main themes of individual psychotherapy with refugees: the importance of recognition and validation within therapy, building a human connection and cultural competence, revisiting trauma and managing difficult emotions, the value of practical interventions, and cultural stigma associated with accessing therapy.

We argue that good mental health and positive social relationships are not only outcomes but also prerequisites for well-being and successful integration in a host country. Aiming to emphasize the refugee’s perspective in understanding the role psychotherapy may play in paving the way for successful integration, we have conducted interviews with refugees who received psychotherapy through the Furchtlos (Fearless) project. In these interviews, we aimed to capture how refugee clients described and evaluated their psychotherapy.

## Methods

Funded by the Foundation Baden-Württemberg, the Fearless project was implemented through a collaboration of the Competence Center for Psychotraumatology of the University of Konstanz, the Lake Constance Institute for Psychotherapy (apb), and the NGO vivo international. The Furchtlos project aims at facilitating refugees’ access to outpatient mental health services. It offers refugees mental health screenings with the Refugee Health Screener [32–34] and referrals to psychotherapy if needed. Refugee clients were recruited for screenings by social workers at the refugee shelters, who informed potential participants, mostly between the ages of 14 and 22, about the project. Inclusion criteria to participate in psychotherapy were the indication for outpatient psychotherapy (e.g., presence of a mental disorder based on the Refugee Health Screener [33]) and willingness to participate in the project assessments. Exclusion criteria were the presence of a mental disorder requiring inpatient therapy (e.g., acute psychosis, acute addiction problems, acute suicidality). Further, to implement an established training plan, psychotherapists in training and their supervisors were trained in using NET; translators, and peer counsellors were trained in guiding the refugee clients through the German health care system. Prior to this study, interviews with the therapists involved in the project were conducted [25].

### Participants and therapeutic modality

Table 1 provides an overview of the demographic information of the six interviewed refugee clients. All of them were male and had regularly terminated psychotherapy. Even though, none of them spoke English or German as their first language, the interviewees were confident enough of their language mastery to conduct the interviews in English (*n* = 3) or German (*n* = 3).

**Table 1:** Demographic related information of interviewees

Category	Count	Category	Count
*Gender*		*Age*	
male	6	18-25	4
		26-35	1
		above 35	1
*Country of Origin*		*Housing Situation at Screening* ^1^	
Afghanistan	1	Government Refugee Center	5
Gambia	3	Private	1
Senegal	1		
Turkey	1		
*Psychological Diagnoses*		*Asylum Status at Screening* ^1^	
PTSD only	2	Current asylum application process	4
PTSD + Depressive Disorder	3	Residence Permit	1
PTSD + Depressive Disorder + Substance Dependency	1	Asylum application rejection; deportation currently suspended	1
*Current Host Country*		*Family in Host Country*	
Germany	5	Yes	1
Other European Country	1	No	5

*Notes*. ^1^Screenings were performed one to two years prior

All therapists were experienced in providing treatments within the German health care system (the majority of them being in training to become licensed Psychotherapists). After receiving training in NET, the therapists chose to take on a refugee client with support from the Furchtlos project. The therapists designed the psychotherapy at their own discretion in accordance with the guidelines in Germany, as part of their therapy five refugee clients had received NET (see Table 2). For more information on the therapists and their view of psychotherapy with refugee clients see Potter [25]. In accordance with research in Germany, psychotherapy was classified as “started” when patients had completed at least six sessions [35, 36]. First project assessments showed that 22% of all psychotherapies classified as started, ended prematurely [25, 37].

**Table 2:** Therapy related information of interviewees

Category	Count	Category	Count
*Received NET as part of their therapy*		*Number of Therapy Sessions*	
Yes	5	17	2
No	1	34-38	3
		50	1
*End of Therapy*			
2021	2		
2022	4		

### Data collection

Qualitative data were collected using a semi-structured interview guide exploring refugees’ psychotherapy experiences. The interview guide, see Supplement A, was developed according to Witzel [38], as a problem-centered interview and also based on episodic interviews according to Flick [39]. The interview guide consisted of open questions about the organizational process of therapy, therapy motivation, therapy content, evaluation of therapy, expectations and notions about therapy, difficulties in therapy, cultural differences, and therapy ending. Potential factors influencing these themes were rephrased into narrative-inspiring questions. Moreover, the interview guide was based on the interview guide used with therapists in a previous study [25] and no pilot interview was conducted. The purposive sampling was an attempted full survey: we attempted to contact all 13 refugee clients who had regularly terminated psychotherapy facilitated by the Furchtlos project via the Furchtlos database or via contacting their former therapists. Ultimately, we had only up-to-date contact details of nine refugees, of whom six responded and agreed to participate in interviews. All interviewees who agreed to participate were contacted and invited to participate in the study by their therapist. Moreover, they were informed in advance about the content of the study, i.e., how they experienced their psychotherapy. Participation was voluntary and interviewees received 50 € for participating. Data collection was completed after six interviews, when all nine potential respondents had been asked to participate. The interviews were conducted by a master student (AR) who did not know the interviewees previously. The interviewer was a 25-year-old Caucasian, non-migrant, German native speaker. She attended research colloquia and several methods workshops on qualitative research methods. Through clinical internships and part-time work, she had professional experience with the psychological treatment of refugees. As a young Caucasian German researcher, AR occupied a structurally more powerful position than the interviewees, not only through the academic role and institutional affiliation with the Furchtlos project, but also as a citizen of the country in which they sought protection. This asymmetry may have influenced what participants felt able or willing to share, particularly regarding negative experiences with psychotherapy or with German institutions. At the same time, the interviews did not remain uniformly positive: many participants spoke about exhaustion, ambivalence, and difficulties in therapy, suggesting that despite potential social desirability, critical experiences were voiced.

The interviews took place between December 2023 and January 2024 and were conducted in person (*n* = 4) or online (*n* = 2). The in-person interviews took place on the premises of the Furchtlos project, and the online interviews were conducted via the BigBlueButton video platform. The interviews lasted between 35 and 90 min, and all interviews were recorded on a voice recorder. Quotes in support of results were translated back and forth from German to English and from English to German in the research team. Before the interviews, the interviewer introduced herself, and the consent form and discussed compensation. All respondents gave their consent for participation, recording of the interview, and further processing, securing, and anonymization of their interview data. An interviewer protocol, completed after the interview, noted special incidents. The interviews were conducted as part of the Furchtlos project, which was approved by the Ethics Committee of the University of Konstanz.

### Data analysis

All interviewees were given pseudonyms, which were used in this study, to protect their privacy. In addition, personal details, location information, and any other identifying data were anonymized. The audio files of the interviews were transcribed by AR using MAXQDA24, first with the help of AI and then manually edited and formatted. The author FP compared the transcripts to the audio recordings and edited them if necessary. After transcription, the audio files were securely deleted. Transcription was based on Kuckartz [40], who developed transcription rules for a computer-optimized literary transcription. To minimize the risk of stereotyping the analysis due to the choice of transcribing language difficulties [41, 42], consultations were held between the authors AR and FP. Furthermore, a summary of the transcript and open coding was sent to the interviewees for review.

The analysis of the interview data was carried out by AR in collaboration with FP. Constructivist grounded theory (CGT) following Charmaz [43] was the applied methodology. CGT builds on the original work on Grounded Theory (GT) of Glaser and Strauss [44] but develops it further by emphasizing the co-construction of meaning as theory building (in contrast to a “discovery” of theory) and reflexivity about researcher and context in the research process. Therefore, all generated qualitative theories are regarded as “versions of the world” [45] constructed by the researcher and research partners [43]. Both data collection and analysis processes were carried out as openly and independently of existing literature as possible, in line with CGT [43]. Extensive literature research was carried out during the middle of the data analysis and during the writing process, to continue reflecting on emerging categories. GT analysis consists of several techniques: open coding, focused coding, theoretical coding, and the use of memos. These techniques guide the researcher from initial data examination to the development of a comprehensive data-explaining theoretical framework grounded in empirical evidence [43]. In this study, line-by-line coding was used to get to know the data. The initial coding of the first three interviews (Ebrima, Samba, Alex) started while the last three interviews (Bassirou, Jawad, Samir) were still being conducted. The code system that emerged from the initial coding helped to navigate the data for subsequent coding steps and notes. In the code system, the data were sorted chronologically, i.e., ‘before therapy’, ‘during therapy’, and ‘after or end of therapy’ corresponding to the way the interviewees told their stories. Specifically, the first phase of focused coding yielded three concepts that are present in the final framework: the therapeutic relationship, opening up, and the guiding role of therapists. Then the data was used to develop different ideas about the relationships between these concepts, moving between open coding, focused coding, and external literature review. When developing the data frame we focused on content either consistently mentioned by (almost) all research partners or identified as new and noteworthy according to Charmaz’s CGT quality criterion of “originality” [43].

### Quality criteria

Flick [46] questioned the applicability of classical quantitative quality criteria to qualitative research and instead described methods of communicative validation, transparency, and justification of validity. Steinke [47] listed indication of the method, empirical anchoring, generalizability, and intersubjective comprehensibility to be used for quality assurance and evaluation in qualitative research. Moreover, Charmaz [43] considered credibility, originality, resonance, and usefulness of CGT as evaluation criteria. To ensure communicative validation [46], consultations were held with the interview partners by sending a summary of each interview to them for confirmation of the content. Further, several consultations were held with other (qualitative) researchers and supervisors: The study was presented at two different points in the research process in two research colloquia at the University of Konstanz and at the Ludwig Maximilian University of Munich, and there were several data sessions and consultations with supervisors to discuss progress and theory development. The COREQ Checklist [48], including elements that should be documented in qualitative research reports, was completed and can be viewed in Supplement B. In the spirit of intersubjective comprehensibility [47] and transparency [46], more information on the research process and coding can be found in Supplement C. Empirical anchoring [47] was achieved by referring to interviewees who made statements about the relevant concepts and also noting contrasting statements. Generalizability was approached by identifying commonalities between the interviewees, reviewing literature, and considering practical implications of the framework [47]. Triangulation [46, 47], i.e., expanding the investigation to include additional researchers, data sets, methods, or theoretical approaches to secure generalizability, proved difficult to achieve in this study as there were no observational data or transcripts of therapy sessions.

## Results

**Fig. 1:**
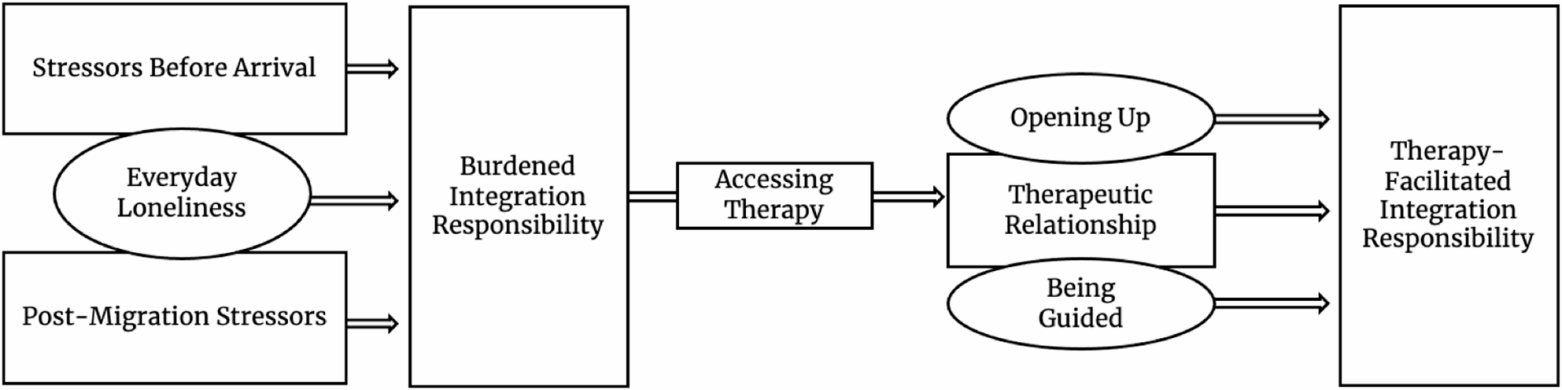
Framework therapy-facilitated integration responsibility

The framework ***Therapy-Facilitated Integration Responsibility*** is presented in Fig. 1. It describes a possible role of psychotherapy for the interviewees in their process of facing past and present challenges related to integration. The framework entails the following concepts: stressors before arrival, post-migration stressors, everyday loneliness, burdened integration responsibility, accessing therapy, therapeutic relationship, opening up, being guided, and therapy-facilitated integration responsibility.

### Burdened integration responsibility

Data regarding pre-and peri-migration stressors is summarized under the term ***Stressors Before Arrival***. Traumatizing experiences were not discussed in detail in the interviews and were usually only roughly named: *“Yes*,* it was a lot*,* because when I was young*,* I left my mother alone*,* came to many countries as a refugee and I had no money and did a lot*,* saw a lot and yes.”* (Translation, Ebrima, ll. 191–192) Rather than talking about distinct experiences, the interviewees shared how they suffered from psychological distress because of them, mainly: reexperiencing and repression of memories, impaired emotion regulation (*n* = 1), concentration difficulties (*n* = 1), social withdrawal (*n* = 2), suicidal thought patterns because of the separation from family (*n* = 1), and substance abuse (*n* = 1): “*[…] when I don’t feel so good and it’s difficult*,* I drank beer. That makes my head feel a little better*,* and then I can stay calm again.”* (Translation, Jawad, ll. 110–111) Samba also described concealed suffering and upholding normalcy:*”Before the therapy*,* like I used to smile a lot. That was my nature. But deep down I was suffering*,* you know*,* so sometimes I feel like I’m losing myself*,* like.”* (Samba, ll. 86–87).

Interviewees mentioned several ***Post-Migration Stressors***: discrimination (*n* = 2), the housing situation (*n* = 4), the COVID-19 pandemic (*n* = 1), language barriers (*n* = 3), unclear residence status (*n* = 3) resulting in frustration (*n* = 1), and being separated from family (*n* = 3). Behind all these categories lie multiple and often intertwined problems: Being separated from family does not only mean missing them, but also being alone in Germany, worrying about them, and may include trying to send them money. Moreover, an unclear residence status may often be closely connected to the housing situation, the opportunity to work or long waiting lists to take part in language courses. All interviewees who were living in Germany at the time of the interviews reported the importance of work (*n* = 3) or education (*n* = 2) in their life. Apart from individual stressors, accumulation and time were mentioned to influence these post-migration burdens. For one interviewee the combination of his insecure residence status, the housing situation, the COVID-19 pandemic and not feeling welcomed in Germany because of his ethnicity, led to suffering, loneliness and *“overthinking”* (Alex, ll. 193):

*“So, you have another stress, another plus, another stress. […] If you sleep at night, if you go to bed at night, will you sleep or not? Because you feel like you are not having life, while you are still suffering. You pass through lot. You come to Germany, maybe you say you want to look for better life, you come, they reject your asyl[um]. So, you are living in condition which you don’t like it, you know. So, people, you go outside also, people look at for your color or your race, you know. So, it causes, a lot of people have mental problem […] because of overthinking. Overthinking can make people easily mad. You know, this is a big problem, a lot of people don’t know why people, a lot, lot of migrants, they feel depressed because of this, because they feel like they are not welcome.” *(Alex, ll. 182–195)

In the interview situation with the, at the time of the interview, deported interviewee multiple post-migration burdens were on full display: he was homeless, receiving food and shelter from a charity organization and trying to return to Germany. Another interviewee only felt happy and relieved for a short time after arrival, then repercussions of past experiences caught up and new challenges came up. Three interviewees further described that, while living in refugee shelters, problems worsened with time, for example because of drug consumption at the refugee shelters (*n* = 1).

While stressors before arrival in a host country may lead to social withdrawal due to psychological symptoms, post-migration stressors may lead to the social isolation of refugees due to the housing situation, cultural adjustment difficulties, racism, and language barriers. In addition, the loss of family and previous social networks can hardly or only very slowly be compensated for in the host country. All interviewees described feelings of loneliness or at least mentioned social withdrawal, isolation, and a lack of relationships in which they could express their emotions and talk about themselves: *“Or sometimes I just feel alone*,* and I have no one to talk to.”* (Bassirou, ll. 384–385) They attributed their loneliness to their depressive symptoms and emotional distress (*n* = 1), their separation from family (*n* = 1), racism, and the COVID-19 pandemic (*n* = 1). The concept ***Everyday Loneliness*** describes this central and profound experience refugees encounter at times even daily before and after migration:

*“So*,* when I came here*,* I was always sitting alone. So*,* all along*,* in my mind*,* I have to talk to somebody*,* like I want to talk to somebody*,* but I don’t know […] I don’t know how therapy works.”* (Samba, ll. 30–32)

In the context of the Therapy-Facilitated Integration Responsibility framework, Everyday Loneliness is another burden on the integration responsibility and influences the therapeutic relationship, the motivation for therapy, and the ability to end it.

***Burdened Integration Responsibility*** reflects the interviewees’ perspective that integration is a process for which they bear sole responsibility:

*“Yeah*,* I think it left for only the person itself […] Like*,* the person him or herself*,* you know*,* to be integrated. I know*,* it’s hard or it’s completely hard*,* when especially*,* when you are different color. But if you want to be integrated*,* you have to meet people.”* (Alex, ll.133–136)

The concept shows the responsibility for the integration process that refugees want but also must take on. Simultaneously, they feel like failing due to their individual burdens, ignoring systemic barriers such as work restriction laws, challenging housing situations or complicated asylum processes in their host country. This perception might explain why one interviewee talked about “*not being able to integrate*” (Ebrima, ll. 6, 31, 67), and why a therapist could change that, despite not changing integration policies in Germany. The interviewees did not use the term responsibility, but in accordance with Charmaz [43] use of theoretical coding in the CGT methodology, this more abstract code works as a bridge in the data. As described in the introduction, integration is a process led by two: Not only the arriving refugees need to adapt, but also members of the dominant culture of the host country must be willing to connect and change. With the concept of Integration Responsibility, it is pointed out that even if the process is supported by the state or members of the host country, several interviewees (*n* = 4) perceived that the responsibility (and therefore the blame if failing this responsibility) was theirs:*“[…]you have to think for yourself how you can integrate into Germany.”* (Translation, Samir, ll. 310–311) Looking at the specific tasks of those involved in the integration process shows that, indeed, the initiation and success of the process must come from the newcomers. The individual has a rather concrete responsibility to integrate, while the host country has a much more abstract responsibility to enable these actions. Refugees must be able to take part in government integration measures, complete language courses, understand how their host country works, and seek contact with possibly xenophobic locals and people of their own culture. Failing to fulfill a perceived integration responsibility could be a result of loneliness and mental health impairments. Hence, we argue that psychotherapy can facilitate the burden of integration through the therapeutic relationship and the processes of Opening Up and Being Guided.

### Accessing psychotherapy

The interviewees initially reported not feeling that psychotherapy was meant for them, because they associated psychotherapy with madness (*n* = 3), dementia (*n* = 1), and stigmatization in their home countries (*n* = 2). Two interviewees also reported that many refugees might fear being questioned in therapy, as it reminds them of questions asked in court or by the police. As reasons for ***Accessing Psychotherapy***, the interviewees mentioned a general interest in therapy (*n* = 2), social workers as important supporters (*n* = 2), or an encouraging friend (*n* = 1). Overall, the interviewees reported different motivations for starting therapy: *“talking to somebody”* (*n* = 2), gaining concentration for school and being able to connect with people without fear (*n* = 1). The Furchtlos project remained the only access point to psychotherapy for all interviewees in this study. They emphasized the outreach aspect of the project (*n* = 4), i.e., conducting screenings in the refugee shelter, as crucial for accessing therapy, as neither knowledge nor motivation were sufficient to overcome the challenges of finding a place for psychotherapy on their own:

*“I started realizing*,* you know*,* if I don’t make any moves or stuff like that*,* if I don’t talk to people*,* it’s going to be so difficult for me. But at that time*,* I don’t know who the right person is to talk to*,* yeah.”* (Samba, ll. 115–117)

### Therapy-Facilitated integration responsibility

Ultimately, the ***Therapeutic Relationship*** played a central role serving as the foundation for therapeutic success but also representing a positive relational contrast to other current or past relationships of the refugees:*“I really liked it because after the first conversation I realized that I have value. Because there are people who think of me and who ask questions and know how I feel. It’s really a good thing*,* I think.”* (Translation, Samir, ll. 175–177) While interviewees mentioned many characteristics of the therapist - such as respectfulness, open-mindedness, patience, and the ability to explain and motivate - for all interviewees kindness and friendliness stood out the most. The experience of kindness and friendliness may have been needed to accommodate the loneliness and counteract experiences of social isolation and withdrawal. For all interviewees (*n* = 6) it did not matter that their therapists were white Germans, and two interviewees emphasized that they needed help no matter from whom. Interviewees also expressed that they perceived their therapists to be open, evaluating and supporting them independently of their cultural background. Moreover, therapists from a different culture could provide cultural comparisons to the host society (*n* = 2). Three interviewees indicated missing their therapists after therapy ended, which illustrates the importance and potential pitfalls of close therapeutic relationships. In conclusion, the therapeutic relationship may serve as the foundation for therapeutic success facilitating the interviewees Opening Up and Being Guided.

The concept of ***Opening Up*** refers to the process of sharing personal information and painful or traumatic memories with the therapist. This process was mentioned as highly significant by all interviewees. Alongside receiving advice from the therapist, it was mentioned as crucial for individual improvement and a positive evaluation of therapy (*n* = 6). Nevertheless, interviewees reported that it was difficult to open up: *“I didn’t really want to talk about my story […] it’s not really easy to talk about yourself with someone.”* (Translation, Samir, ll. 78–79) Several reasons were mentioned, why it was difficult to talk about personal matters with the therapist, including trust issues (*n* = 1) and fears associated to privacy and potential leakage of information, possibly to governmental institutions involved in the asylum process (*n* = 4):

*“Maybe some people think about like that*,* she is interviewing me*,* my asyl[um]. Maybe she is working with police*,* maybe she is working with*,* everybody can have different imagination or thinking*,* because if you are asyl*,* you always think*,* you don’t trust anybody.”* (Alex, ll. 335–337)

While the association with government came up several times (*n* = 3), three interviewees emphasized more personal reasons for privacy, for example, fear of bringing the past back to life (*n* = 1). Four interviewees reported that answering questions in therapy about their past had been painful, both emotionally (*n* = 4) and physically (*n* = 2):

*“[…] whenever I talk about my story*,* you know*,* it gave me a lot of headache because I cried a lot during the therapy*,* so starting was wasn’t easy*,* definitely. Yeah so*,* I even remember sometimes when I finished therapy*,* then in my mind I would say okay next week I’m not going to come.”* (Samba, ll. 52–54)

Two main ways of talking about sharing were noticeable: Opening Up Despite captures the act of disclosure despite pain and challenges. Confiding in someone new, despite being traumatized and alone for a long time and despite it hurting a lot, required a leap of faith from the interviewees. For this leap, the therapist had to create the right conditions and explanations (*n* = 6). Even though opening up was described as painful, the interviewees continued to share their stories and feelings. The reason may lie in the second narrative of opening up: Finally Opening Up highlights participants’ retrospective sense of having ultimately found someone to confide in and the subsequent emotional experience of relief and connection when finally sharing their story. Thus, Finally Opening Up describes the feeling of finally finding someone to talk to about oneself, about personal challenges (*n* = 4). As shown in the concept of Everyday Loneliness, social isolation and social withdrawal prevented interviewees from experiencing meaningful and trusting relationships. A strong therapeutic relationship, combined with being motivated but not pressured to talk, created an atmosphere in which interviewees wanted to open up (*n* = 3):

*“They give me also the opportunity*,* you know*,* to open myself*,* you know*,* to say what’s happened in reality*,* you know. And to*,* okay*,* to talk to someone. And it’s give me*,* yeah*,* I don’t know how to explain it*,* uh*,* I feel good*,* you know*,* to express it.”* (Bassirou, ll. 15–17)

Moreover, the interviewees valued receiving advice and ***Being Guided*** in the process of managing the past and present. The data suggests that the therapist was seen as someone who could provide answers to the interviewees’ questions and put their ruminations to rest (*n* = 5). Four interviewees noted that only a professional therapist could offer them good advice. While the content of the advice was not always mentioned, it may often have been related to traumatic memories, insecurities and day-to-day challenges. Jawad underlined the implications of receiving advice and the process of accepting it:

*“And so*,* they talk and from these talks you realize*,* ah*,* okay*,* that’s good*,* that’s not*,* that’s normal*,* that’s that. And you have to collect a few words*,* for example*,* keep them in your head and so on. Yes*,* and you have to accept these words or follow them or so on. And that brings your head back to clarity.”* (Translation, Jawad, ll. 209–214)

Some interviewees emphasized that it is usually parents who guide one through life (*n* = 3) but now they are absent, precisely when advice and guidance are most needed. Assuming a parent-like role, offering full attention, personal advice, and guidance through the challenges of trauma processing and integration in Germany may be a key role that therapists, perhaps even unknowingly, come to fulfill.

This psychotherapeutic process was perceived as very helpful (*n* = 6) and led to a **Therapy-Facilitated Integration Responsibility** reflecting the enhanced ability of refugees to take on integration tasks and fulfill the perceived Integration Responsibility after therapy. It seems that therapists temporarily shared their clients’ perceived integration responsibility, thereby making it easier to fulfill: The therapist approached their client in a friendly manner (counteracting loneliness and social isolation), supported the processing of traumatic experiences (counteracting social withdrawal and other trauma-related symptoms), and assumed a guiding role in the acculturation process and integration responsibilities (counteracting helplessness).

*“Everyone wants to earn money*,* live well*,* buy a car*,* buy a house. But if you don’t have a sound mind*,* how can you do that? It would always be difficult. And that’s why I appreciate your work. And yes*,* I am always happy to come here*,* because I know that you have helped me a lot. I still make mistakes*,* I am human. But now I understand.”* (Translation, Ebrima, ll. 318–322)

Six areas of improvement, each contributing to the interviewees’ ability to take on integration actions and enhance well-being, were identified in the data: the reduction of psychological symptoms (*n* = 5), the acceptance of the past and its remaining impairments (*n* = 4), better dealing with (traumatic) memories (*n* = 3), better social skills (*n* = 3), (future-oriented) structuring of everyday life (*n* = 2) and higher self-worth (*n* = 2):

*“The surprise is that I now have a good life. Yes*,* now I can interact with many people*,* I can fall in love and be happy*,* which I couldn’t do before. I used to have long hair and didn’t care about my appearance. I would go out without paying attention to how I looked. I didn’t see myself in the mirror*,* didn’t know how I looked. But now*,* I can look in the mirror*,* see how I look before going out*,* and comb my hair. Before*,* I couldn’t do any of this.”* (Translation, Ebrima, ll. 142–146)

Interviewees reported being better able to meet their academic (*n* = 2) or job requirements (*n* = 1), for example by suffering less from concentration difficulties (*n* = 1) or being ill-tempered (*n* = 1). At the same time, some interviewees emphasized that psychotherapy alone could not solve all of their problems and that personal effort remained essential:

*“I also said before that therapy doesn’t help 100%. Yes*,* maybe 50 to 70%*,* 60% helps*,* because when you hear something from people*,* it shows you a way*,* then you have to follow that way*,* and that was okay*,* yes. And it helped me about 50%.”* (Translation, Jawad, ll. 69–71

For one interviewee, it was difficult to identify specific improvements, although he rated the therapy as very helpful for himself and others. A possible reason could be that he was forced to leave Germany due to his residence status. Unlike the other interviewees, he currently had no perspective of remaining in Germany, rendering integration efforts impossible.

## Discussion

Interviewees reported experiencing significant stressors both prior to and after migration—particularly everyday loneliness—which hindered their ability to fulfill the integration tasks they perceived as their individual integration responsibility, such as learning German, securing employment, and forming social connections. In therapy, therapists adopted a friendly and supportive approach, helped clients process traumatic experiences by encouraging them to open up, and guided them throughout the acculturation process. As a result, interviewees described feeling empowered after therapy to engage in integration efforts they saw as their own responsibility, leading to a Therapy-Facilitated Integration Responsibility. This study extends existing research on psychotherapy with refugees [28, 30, 31, 49] by integrating therapeutic factors into an explanatory model of a perceived Integration Responsibility. In this study, participants described an individual integration responsibility which relates to research in which refugees reported feeling a pressure of integration which may be “a normal kind of stress as newcomers in the process of migration.” ([50], p. 6, l. 5–6). As found in Phillimore [19], many interviewees described feeling unable to meet integration expectations. Their perceived “failure” included not being able to focus on their German classes, to start planning their future, or to overcome social isolation and loneliness. Consistent with prior literature [3–5, 23, 51], integration challenges were influenced by psychological burdens stemming from stressors experienced before and after their arrival. Furthermore, the results of this study emphasized the experience of loneliness as a factor profoundly contributing to a **Burdened Integration Responsibility**, i.e., the perception of solely being responsible for integrating successfully into the host society whilst being overwhelmed with emotions related to the past and left alone with challenges in the new environment. Through accessing therapy and being offered a stable therapeutic relationship, the refugees experienced, often for the first time since arriving in Germany, the possibility to open up and receive guidance within the host society. The refugees emphasized this experience as crucial to transform their burdened sense of integration responsibility into a shared Therapy-Facilitated Integration Responsibility.

### Clinical implications

In addition to suffering from psychological burdens due to the experience of stressors before arrival and post-migration stressors [2, 52, 53], **Everyday Loneliness** has been highlighted as a key issue amongst refugees (Strijk et al., 2011). Research has shown a well-established connection between social isolation and refugees [4, 20, 27, 29, 31, 54]. Loneliness has been identified as an international public health threat, and is an important issue in many psychotherapies [55]. For therapists working with socially isolated and withdrawn clients, especially, but not only refugees, “treating loneliness” might be an unarticulated but immediate expectation [56]. However, it is a rather neglected phenomenon in psychotherapy research and practice, and further guidance might be needed [56–58]. Meanwhile, the role of psychotherapeutic relationships in alleviating everyday loneliness in refugees remains a promising but underexplored area, warranting further investigation.

The **Therapeutic Relationship** plays a central role in the healing process and is an important factor for therapy success, regardless of theoretical school or individual technique [59, 60]. While the importance and challenges of the therapeutic relationship are not specific to refugee clients, it can be argued that given the significant role of loneliness and ongoing migration stressors establishing a safe and stable therapeutic relationship is particularly crucial. Bowlby [61] viewed the therapeutic relationship as a form of attachment, highlighting thereby its importance for clients suffering from attachment or relational disorders and interpersonal and complex trauma. Building a trustful therapeutic relationship with traumatized clients has been reported as challenging [62–66]. Nevertheless, the interviewees indicated that while it was hard to disclose and trust, it might also be something that refugee clients or anyone experiencing Everyday Loneliness longs for. The importance of a therapeutic relationship when working with refugee clients is evident in research [27, 30, 31, 67–69]. In their meta-synthesis, Khairat [31] showed that one of the most present themes in qualitative psychotherapy studies with refugees was the feeling of a human connection to the therapist and feeling validated and welcomed. As our interviewees described, the therapeutic relationship can become very close and even reminiscent of the relationship with one’s own parents or friends [28, 29, 70]. In contrast to the findings of Al-Roubaiy [49], the interviewees in this study did not mention culturally insensitive treatments. The therapists’ origin did not play a major role for them. These findings may have resulted from training all therapists beforehand within the Furchtlos project and from them reporting to be motivated to work with refugee clients [25]. Moreover, while therapists did not describe feeling responsible for the integration demands perceived by their clients, they reported adjusting their expectations and the pace of therapy to the clients’ needs and capacities, demonstrating an understanding of their situation and responding flexibly to structural obstacles related to integration challenges [25]. This sensitive balancing between treatment goals and integration-related challenges may have contributed to alleviating clients’ perceived burden of integration responsibility.

The necessity of a strong therapeutic relationship becomes even more evident when considering the challenge of **Opening Up**, especially regarding traumatic memories. Talking about traumatic events has been mentioned as difficult by unaccompanied refugees receiving NET [30] and as draining and tiring by clients receiving CBT [71]. The pain of disclosing suppressed and traumatic memories and trusting seems to be a common experience [71–73], which is also reported by psychotherapists treating refugees [27]. Similar to our results, the therapists’ role in creating trust, and providing safety to encourage patients’ engagement in reliving has been emphasized as highly relevant [71]. Given the challenges of loneliness, potential traumatization, and individual and structural barriers, the role of therapists in this framework often extends beyond mere listeners to becoming active guides, as reflected in the concept of **Being Guided**. Advising clients has been reported as a common procedure in therapy [74] and may even be expected by clients, especially those with a lower socio-economic status or a cultural background where doctors and therapists often represent authorities [75–77]. Due to the duration and specific requirements for psychotherapy training in Germany^1^, it can be assumed that psychotherapists working in Germany have, if they have not grown up here, at least lived in Germany for a long time. Hence, for new arrivals, they offer a potential point of contact and guide for navigating the host country.

### Integration outcomes

The role of psychotherapy in supporting refugees to fulfill their perceived integration responsibilities is explained by the reduction of trauma-related symptoms and by the development of interpersonal skills, such as trust, disclosure, and receiving advice.

The **Therapy-Facilitated Integration Responsibility** framework further indicates that therapy’s effects extended beyond symptom relief, enhancing refugees’ ability to meet socially and politically required integration tasks. Consistent with qualitative research [27, 30, 31, 67], our results highlight therapy’s potential to support refugees in taking responsibility for their integration. However, this framework does not claim that therapy guarantees successful integration as it cannot remove structural barriers; rather, it offers a needs-oriented, individual approach, where therapists may share their clients’ integration responsibility for a limited time. The finding that participants conceptualized therapy success partially through their enhanced ability to engage in integration measures may reflect a strong pressure of societal discourse in Germany on refugees to prove their worthiness [16]. While psychotherapy was experienced as a space for personal healing, it simultaneously became a means to fulfill societal expectations of being “well-integrated” pointing to underlying societal power dynamics, where refugees’ psychological recovery may be intertwined with structural pressures to integrate. While the present findings refer to psychotherapy in general terms, it is important to note that most participants had received NET. NET’s structured focus on constructing a coherent life narrative may have shaped particularly the processes of Opening Up and Being Guided. The framework of Therapy-Facilitated Integration Responsibility may, at least in part, reflect therapeutic mechanisms specific to trauma-focused, narrative-based approaches. At the same time, the strong emphasis participants placed on the therapeutic relationship suggests that the overarching concepts extend beyond NET to other modalities.

Despite a high need for psychotherapy, only a few refugees are admitted to adequate treatment in Germany [7, 78, 79]. During the first 36-months, most asylum seekers in Germany do not have regular health insurance and additional services, such as psychotherapy, must be approved on a case-by-case basis [80]. Moreover, a general mental health screening for all arriving refugees has not yet been implemented despite European guidelines and research recommendations [81, 82]. Furthermore, therapists reported barriers such as organizational difficulties, including lack of adequately trained translators and funding for them, as well as insufficient expertise in dealing with (traumatized) refugees and cultural differences [25, 83–85]. Especially a high bureaucratic effort was shown to reduce therapists’ future motivation for treating refugee clients [25]. Refugees further reported barriers such as language challenges and, missing information about the German health care system and mental health services [86, 87]. Satinsky et al. [88] reported in their systematic review additional barriers such as stigma, different help-seeking behaviors, lack of awareness, and negative attitudes towards and by providers. Several of these barriers were found in the present data: interviewees mentioned stigmatization, to not have initially felt that the offer of psychotherapy was meant for them, and to fear being questioned in therapy. In contrast to research showing that refugees’ access to psychotherapy needs to be additionally supported and simplified, refugees’ access to health care in Germany has recently been further restricted [89]. Studies have shown that the dissemination of effective treatment to personnel, even with limited training can substantially improve mental health challenges within the community [90–92]. To address the gap in mental health care and integration support a screen-to-treat, stepped-care trauma rehabilitation program has been proposed where refugees are offered three different levels of support after a personal interview: attentive waiting, migrant counsellors delivering Narrative Trauma Counselling (NAT), adapted from NET [93], and treatment by a licensed psychiatrist or psychotherapists for more complex cases [81].

### Limitations

The biggest methodological limitation is the number of interview partners. According to Charmaz [43], the number of interviewees is determined by theoretical saturation, i.e. when no more relevant information is added through new interviews. In this study, the number of interviewees was determined by whether the potential interviewees responded to the interview request, which six out of 13 did. Moreover, all six interviews were conducted in a short time frame, so there were no interviews after the final concept had been developed. The iterative nature of the GT methodology was therefore only followed within the data analysis, not during data collection. If more interviewees had been available, it would have been enriching to collect new data to confirm the framework or find contradictions. One possible reason for the low number of interviewees may be that interviews were held one to two years after the therapy had ended. Although this long delay constitutes an advantage of this study reflecting on refugees’ long-term perspective on therapy, it most likely impeded the participation of several potential interviewees. Within marginalized client groups, it can be difficult to obtain a big enough number of interviewees, and an average experience may be less common in psychotherapeutic practice with minority clients [94, 95]. As the sample is small, all male, and recruited by therapists, there may have been a selection bias resulting in a positive-outcome and social-desirability bias. Iraqi men in Sweden were interviewed on their experiences of counselling/psychotherapy and reported several difficulties with their therapists, including culturally insensitive treatment or not feeling understood [49]. While in this study no negative therapy experiences were reported, any conclusions drawn from the framework must take possible negative experiences into account. Especially as we could not interview all clients who had ended their therapy in the Furchtlos project or clients that dropped out of psychotherapy. While the sample includes only males and was rather homogeneous regarding housing situation and time passed since the therapy had ended, the interviewees came from different countries and had most probably had different experiences in both their home countries and in Germany. According to the GT method, ‘Therapy-Facilitated Integration Responsibility” is a data-explanatory theory and hence not necessarily generalizable. While the presented references to literature point to a plausible generalizability of findings, this framework is one of multiple possible interpretations that apply to the available interview data reflecting subjective experiences in their complexity. A too strong generalization would not do justice to the different situations, resources, and personalities of refugees in Germany [96]. Power-related dynamics between the researchers and the interviewees were reflected on by including the interviewees in the analysis by reporting back an interview summary and all interviewees agreed with their interview summary. Further, social desirability was reflected on by ensuring that the interviews were conducted by a master student who did not know the interviewees previously. Nonetheless, psychotherapy cannot be conceived outside of structural inequalities and therapists, especially those treating minorities, must be aware of those dynamics.

## Conclusions

Psychotherapy emerged as a crucial support mechanism for the interviewees, assisting them in managing migration stressors, feelings of loneliness, and psychological symptoms caused by traumatic experiences. The therapeutic relationship facilitated the process of opening up, sharing personal thoughts, and receiving valuable guidance. As a result, the interviewees found it easier to build and maintain social relationships, concentrate at school, deal with interpersonal and stress-related challenges at work, stop maladaptive coping strategies such as addiction, and cope better with memories and worries about family members. The concept of Therapy-Facilitated Integration Responsibility underscores the significant role of therapy, especially trauma-focused and narrative-based approaches, in easing the integration process and overall well-being. It underlines the importance of facilitating refugees’ access to mental health services. Ultimately, integration is a collaborative process in which the refugees’ responsibility needs to be supported, amongst others also by psychotherapists reinforcing refugees’ personal resources for integration. We conclude that mental health support should be incorporated into integration policies to enhance refugees’ integration, benefiting both the individuals and the host society.

## Supplementary Information

Below is the link to the electronic supplementary material.

Supplementary Material 1

Supplementary Material 2

Supplementary Material 3

## Data Availability

The raw data supporting the conclusions of this article will be made available by the authors, without undue reservation.

## Footnotes

1 To become licensed psychotherapists, psychologists in Germany complete a three-year training after getting their master’s degree.
