# Supplementary material for: Therapy-facilitated integration responsibility: qualitative interviews with refugee psychotherapy clients in Germany
Source: BMC Psychiatry. 2026 Jan 12;26:82. doi: 10.1186/s12888-025-07716-0 (PMC12849516; doi:10.1186/s12888-025-07716-0)
Supplement: Supplementary file 1 — Supplementary Material 1 [file 12888_2025_7716_MOESM1_ESM.docx]

# **Supplement A**

**Table 2.***Interview Guideline*

| Category | Narrative Prompt | Follow-up and Rephrasing Options^1^ |
| --- | --- | --- |
| Introduction | (small talk, informed consent, introduction researcher) |  |
| Organizational Process of Therapy | How did you manage to fit therapy into your everyday life? | *Did you find it easy to keep up with the appointments?* |
| Therapy Motivations | Why did you start going to psychotherapy? | Can you tell me about a situation in which you thought: I want to do psychotherapy now? |
| Therapy Content | Tell me about your therapy! What did you do? |  |
| Evaluation of Therapy | What does it feel like to do psychotherapy? | *How did it feel to talk so much about your own life?  Were there times when you didn't want to go to therapy?  Or times when you really wanted to?* |
| Expectations and Notions about Therapy | What surprised you most about psychotherapy? | Which expectations were fulfilled, and which were not? *What do you think about mental illnesses today?* |
| Difficulties in Therapy | What is difficult about doing psychotherapy? | Can you tell me about a difficult situation in therapy? *What do you think prevents other refugees from going to therapy?* |
| Cultural Differences | What do you think about coming from a different country than your therapist? | What does this mean in therapy?  Example: Negative: the therapist doesn't know the country and therefore doesn't understand what I'm saying. I'm worried about what the therapist thinks. Positive: The therapist is very interested and aims to understand me.  Has there been a situation in which your origin has influenced your therapy? How did you and your therapist deal with it? Would it have been easier if you had had a therapist from your country? |
| Therapy Ending | How did your therapy end? | *What do you take away from therapy for your further life?* Was there anything else you would have wanted or needed from the therapy or the therapist? |
| Interview Ending | Would you recommend psychotherapy to other people after your experience? | Is there anything else I should know about psychotherapy? |

Notes. ^1^ Questions in italics were to be asked if they had not already been answered by responding to initial narrative prompts.
