# Supplementary material for: Therapy-facilitated integration responsibility: qualitative interviews with refugee psychotherapy clients in Germany
Source: BMC Psychiatry. 2026 Jan 12;26:82. doi: 10.1186/s12888-025-07716-0 (PMC12849516; doi:10.1186/s12888-025-07716-0)
Supplement: Supplementary file 2 — Supplementary Material 2 [file 12888_2025_7716_MOESM2_ESM.docx]

# **Supplement C**

This section describes the coding and analysis processes of GT as postulated by Charmaz (2014) and the resulting emergence of inductive categories and concepts. GT analysis consists of several techniques: the use of memos, open coding, focused coding, and theoretical coding. These techniques guide the researcher from initial data examination to the development of a comprehensive theoretical framework grounded in empirical evidence (Charmaz, 2014). The aim of this section is to provide insights into the coding process and to approach the theoretical construct of the present study.

**Memo Writing**

For a successful coding process, Charmaz emphasized the importance of writing memos. Memos are reflective notes that researchers write to document their thoughts, insights, and analytical decisions (Charmaz, 2014). Charmaz described them as an ongoing dialogue with oneself about the data and the emerging analysis; they help in elaborating on codes, exploring theoretical ideas, and making connections between categories. This practice ensures that the analytical process remains dynamic and evolves with the data (Charmaz, 2014). So, throughout the analysis and especially during the open and focused coding, a research diary was kept in which thoughts about the data after each day of analysis were written down. At the beginning of the coding process, the notes were mostly describing the data and considering possible topics for the theory. As the analysis progressed, the memos became less personal and more topic-based, putting forward hypotheses and questions regarding directions of the theory. At the end of the analysis, the memos were supplemented with literature and formulated in relation to the concepts developed in focused coding.

**Initial Coding**

In Charmaz's (2014) GT, initial or open coding is a first step in the coding process: At this stage, the data is examined line by line or event by event to identify relevant words, phrases or sections, and these are mostly labelled descriptively. The aim of initial coding is to develop initial categories in order to generate as many ideas as possible without drawing premature conclusions (Charmaz, 2014). Charmaz emphasized that the analysis remains closely tied to the data, indicating the importance of open coding. It is a reflective process in which the researcher engages deeply with the data, fostering insight and interpretation (Charmaz, 2014). It sets the tone for the analysis that follows, but as with all coding steps in GT, it is part of an iterative process. In this study, line-by-line coding was used to get to know the data. The initial coding of the first three interviews (Ebrima, Samba, Alex) was started while the last three interviews (Bassirou, Jawad, Samir) were conducted. With each new interview similarities, differences or completely new information were recognized quicker and codes could be reused. However, the research diary and frequent use of in vivo codes helped to maintain a degree of impartiality in the coding of later interviews. Table 1 shows an example of initial coding.

| **Table 1** | |
| --- | --- |
| *Examples of Initial Coding* | |
| Codes | Conversation Excerpt |
| afraid to share past reflecting past experiences wanting to share afraid or ashamed how can I express it?  depressive symptoms being at home alone past comes automatically improvement  dealing with thoughts couldn’t talk to neighbors past is absorbing not able to connect social when repression | *yes, at the beginning i was still afraid because i didn't want to share my story with anyone(.) what have i seen(?) what have i done(?) because it was a lot, and i was young and, yes, i wanted to say it but i was still afraid to share it with someone: what have i experienced, what have i done(.) and it was very, very bad for me(.) how can i express it(?) when you hold onto it, it’s not good for you, it depresses, you get depression(.) and i held onto it and couldn’t let it go because it stayed with me(.) and sometimes, when i’m at home, bored, when i’m home alone, then you think about the past(.) what comes, it comes automatically(.) when i’m alone, quiet, it comes automatically(.) but now it has improved a lot(.) but sometimes it comes, but now i know how to get it away from me(.) but before, when it was depressing, then i was stressed, i couldn’t talk to my neighbors because i became depressed, i was stressed(.) what did i experience before, what did i do, then i was stressed(.) then i couldn’t talk to anyone the whole day(.) if it goes away from me, then i could go out, could go to people and communicate with people, yes(.) (Translation, Ebrima, 106-117)* |

The code system that emerged from the initial coding helped to navigate the data for subsequent coding steps and notes. Like the open codes, it is descriptive. In the code system the data were sorted chronologically, i.e. 'before therapy', 'during therapy' and 'after or end of therapy'. This temporal structuring corresponded to the way the interviewees told their stories and helped to find data for concepts that were developed later in the analysis.

**Focused Coding**

Focused coding follows initial coding and involves selecting the most significant and frequent initial codes to categorize large data segments (Charmaz, 2014). According to Charmaz, this stage is more focused and selective, aiming to refine and synthesize the data into fewer, more coherent categories. Researchers use this stage to determine which initial codes best capture the patterns in the data; focused coding helps to clarify emerging categories, setting the stage for more abstract analysis (Charmaz, 2014). Table 2 presents an interview excerpt with some codes developed in a focused stage of coding.

**Table 2.***Examples of Focused Coding*

| Codes | Conversation Excerpt |
| --- | --- |
| Opening Up: Time Demands  Opening Up: Ambivalence Responsibility for Past Opening Up: Letting Go  Loneliness: Boredom  Loneliness: Amplifying Change: Ability to Act  Social Withdrawal | *yes, at the beginning i was still afraid because i didn't want to share my story with anyone(.) what have i seen(?) what have i done(?) because it was a lot, and i was young and, yes, i wanted to say it but i was still afraid to share it with someone: what have i experienced, what have i done(.) and it was very, very bad for me(.) how can i express it(?) when you hold onto it, it’s not good for you, it depresses, you get depression(.) and i held onto it and couldn’t let it go because it stayed with me(.) and sometimes, when i’m at home, bored, when i’m home alone, then you think about the past(.) what comes, it comes automatically(.) when i’m alone, quiet, it comes automatically(.) but now it has improved a lot(.) but sometimes it comes, but now i know how to get it away from me(.) but before, when it was depressing, then i was stressed, i couldn’t talk to my neighbors because i became depressed, i was stressed(.) what did i experience before, what did i do, then i was stressed(.) then i couldn’t talk to anyone the whole day(.) if it goes away from me, then i could go out, could go to people and communicate with people, yes(.) (Translation, Ebrima, 106-117)* |

Charmaz (2014) explained that the analytical-explanatory level of codes can be enhanced through focused coding. Focused coding occurred relatively late in the analysis process of this study, before that most of the time was spent systematizing and organizing the codes. After sorting the content, the aspects that seemed most interesting and recurrent were focused on. During this process, the focus on the data and the naming of concepts often changed. For example, "Opening up" was temporarily referred as "Exposition" to highlight the therapeutic technique involved, but then words and perspectives of the interviewees prioritize. Similarly, initially the guidance function was viewed solely through the lens of a family substitute, and while it still plays a role in the current framework, it now addresses a more realistic function as described by the interviewees rather than the therapist being a substitute parent.

Specifically, the first phase of focused coding yielded three concepts that are also present in the final framework: the therapeutic relationship, opening up, and the guiding role of therapists. Then the data was used to develop different ideas about the relationships between these concepts, moving between open coding, focused coding and external literature review.

**Theoretical Coding**

Charmaz (2014) postulated that theory building can be effectively accomplished with iterative open and focused coding, ensuring the theory remains grounded in the empirical data. Yet they mentioned theoretical coding as an additional step to add more depth to the concepts and resulting theory. Theoretical codes are codes constructed to connect and explain existing codes and concepts better (Charmaz, 2014). They are less data-connected than focused codes, so Charmaz cautions that theoretical coding carries risks, such as imposing preconceived ideas onto the data. Although theoretical coding was never practiced as a separate phase from focused coding, this technique was often used in the analysis. For example, “Integration Responsibility” was not explicitly mentioned by the interviewees, it was developed as a bridging concept. There is data to suggest that respondents feel integration responsibility, but it remains a theoretical construct that brings together the findings of the other concepts. Although theoretical coding according to Charmaz (2014) does not have to be part of GT, it has helped to develop an abstractable, generalizable but still data-related framework.

**Earlier Theory Drafts**

In order to increase the transparency and traceability of the analysis process, in addition to the content coding system, two earlier drafts of a theoretical construct are also shown. The first framework, figure 1, like the final result, is rather linear, but has neither the concept of integration responsibility nor the description of migration stressors. The idea behind it was that integration is a very complex and often negatively connotated concept, instead "increased wellbeing in host country" was chosen as the outcome. This would also be applicable to the concept of "therapy-facilitated integration responsibility". However, it was ultimately decided that integration can be used both as an emic term of the interviewees and because of its importance in the related body of knowledge and policies. The second draft, Figure 2, represents a less linear draft that was developed as a precursor to the final framework. Here it is clear that the content is arranged in a more hierarchical manner, which can also be deduced from the fact that the focused coding was more advanced.

**Figure 1.** Theory Draft 1


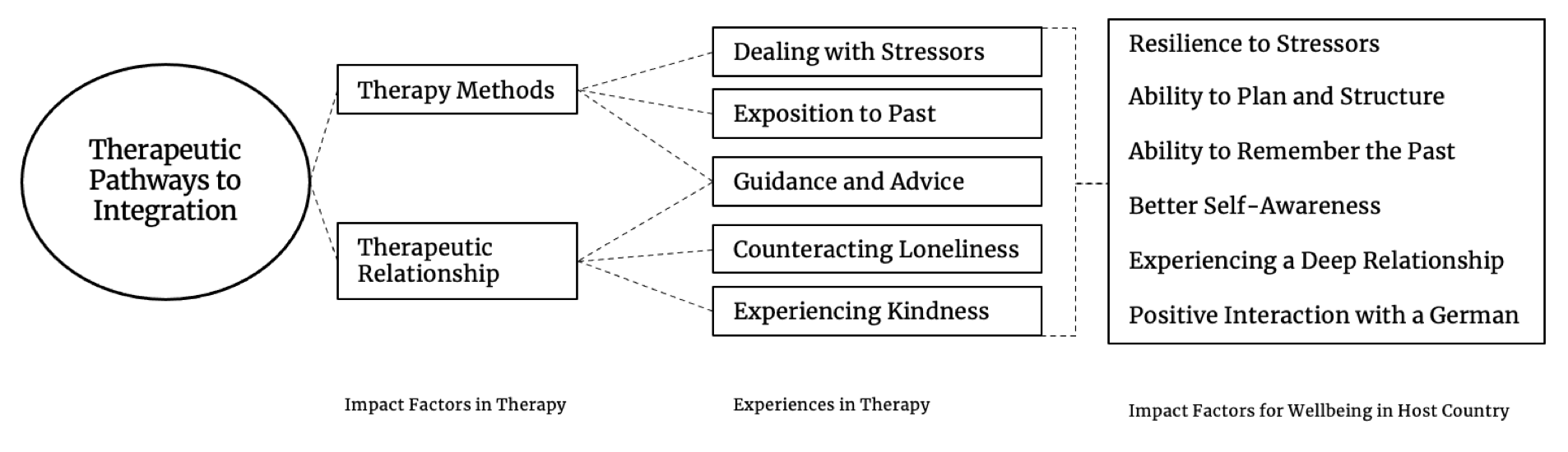


**Figure 2.** Theory Draft 2


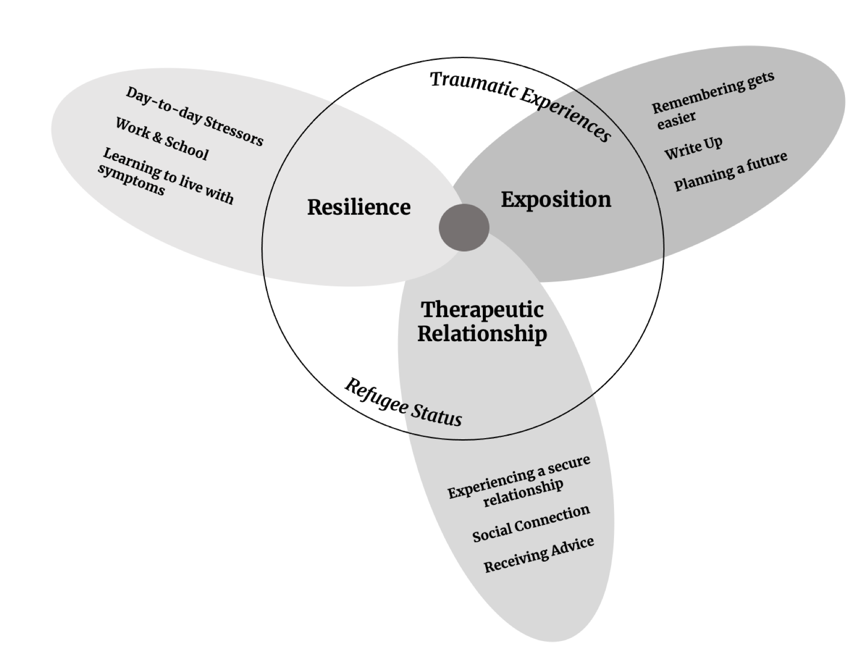


**References**

Charmaz, K. (2014). *Constructing Grounded Theory (Introducing Qualitative Methods series)*. sage.
